# Diagnosis of massive obscure gastrointestinal bleeding from a Dieulafoy lesion identified by intraoperative enteroscopy

**DOI:** 10.1055/a-2528-6967

**Published:** 2025-03-25

**Authors:** Jiaqi Gao, Tianyu He, Liping Duan, Rongli Cui, Zhijie Xu

**Affiliations:** 1 66482 Department of Gastroenterology, Peking University Third Hospital, Beijing, China; 2 Department of Pathology, Peking University Third Hospital, Beijing, China

A 32-year-old woman was admitted to the emergency department with syncope, shock, and persistent hematochezia. Her hemoglobin had drastically declined from 14.1 g/dL to 5.5 g/dL within 4 hours. Prompt, although challenging, central venous catheterization coupled with rapid intravenous fluid resuscitation elevated her systolic blood pressure from 60 mmHg to approximately 90 mmHg. Computed tomographic angiography (CTA) revealed a dense shadow in the bowel lumen in the left upper abdomen ( Fig. 1 ). Consequently, a multidisciplinary team collaborated to perform an open laparotomy, complemented by intraoperative endoscopy.

**Fig. 1:**
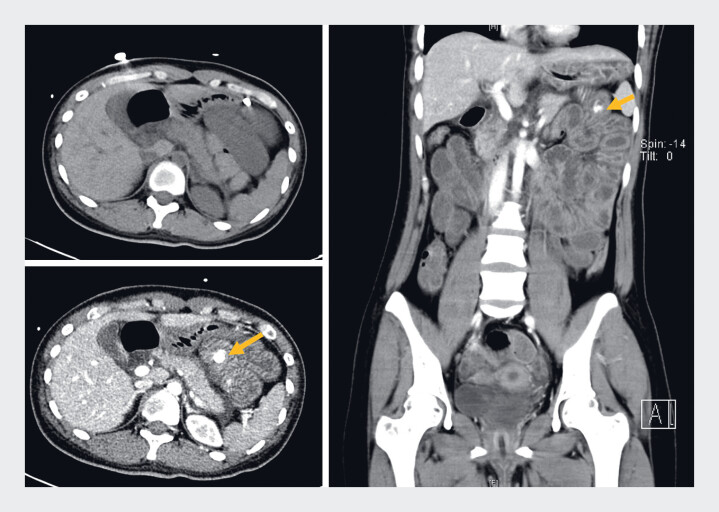
Computed tomographic angiography revealed a dense shadow (arrows) in the bowel lumen in the left upper abdomen.

During the intraoperative endoscopy, a hemispherical mucosal protrusion, approximately 0.8 cm in size, was identified in the upper jejunum ( Video 1 ). The mucosa’s surface was smooth, and a jet-like hemorrhage emanated from its apex. Endoscopic titanium clip occlusion was performed to stop the bleeding, followed by surgical resection of this intestinal segment ( Fig. 2 ).

Intraoperative enteroscopic identification of the source of massive obscure gastrointestinal bleeding.Video 1

**Fig. 2:**
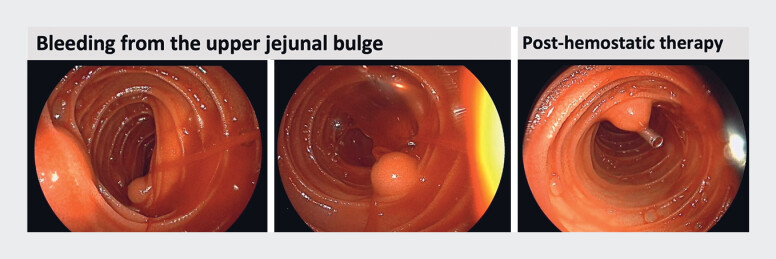
Intraoperative endoscopy showed a distinct hemispherical mucosal protrusion in the upper jejunum. The mucosa’s surface was smooth, and a jet-like hemorrhage emanated from its apex. Endoscopic titanium clip occlusion was performed to stop the bleeding.

Subsequent histological examination revealed a focal thick-walled, lumen-dilated artery with localized hemorrhage within the submucosal layer of the small intestine, exhibiting morphological features consistent with constant-diameter arteriopathy, known as Dieulafoy disease ( Fig. 3 ).

**Fig. 3:**
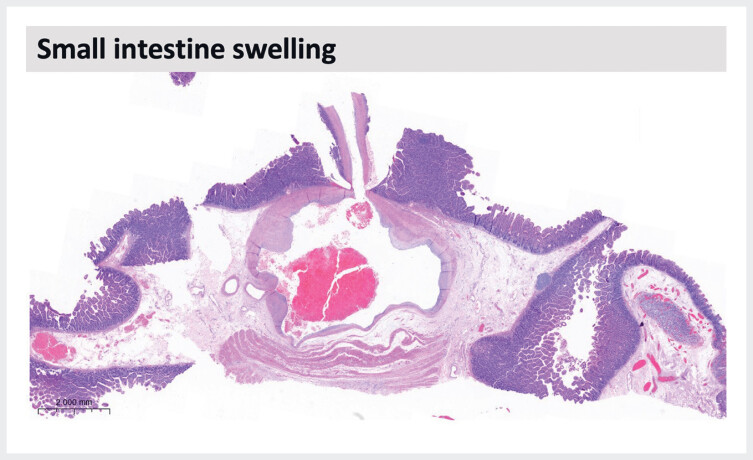
Histological examination revealed a Dieulafoy lesion.

Intraoperative endoscopy, angiography, and indeed device-assisted enteroscopy remain the recommended first-line modality for treating hemodynamically unstable patients with catastrophic obscure gastrointestinal bleeding, despite the rapid technological advances in capsule endoscopy and emergency enteroscopy 1 2 . Intraoperative endoscopy is more accurate and effective for small-bowel bleeding than interventional angiography, as the latter can be challenging where there is a slow bleeding rate, difficult vascular access, or extensive collateral circulation, and it also carries a relatively high complication rate 3 . For patients with brisk bleeding, CTA is favored as a noninvasive tool to identify the potential source and determine the optimal timing for intervention, thereby facilitating a prompt and precise intraoperative endoscopy plan 4 . Furthermore, this case exhibited a rare clinical feature of a Dieulafoy lesion which, unlike most reported Dieulafoy lesions, mimicked a stromal tumor during the acute hemorrhagic phase 5 .

Endoscopy_UCTN_Code_CCL_1AC_2AB

## Notes

Endoscopy E-Videos https://eref.thieme.de/e-videos
*E-Videos* is an open access online section of the journal *Endoscopy* , reporting on interesting cases and new techniques in gastroenterological endoscopy. All papers include a high-quality video and are published with a Creative Commons CC-BY license. Endoscopy E-Videos qualify for HINARI discounts and waivers and eligibility is automatically checked during the submission process. We grant 100% waivers to articles whose corresponding authors are based in Group A countries and 50% waivers to those who are based in Group B countries as classified by Research4Life (see: https://www.research4life.org/access/eligibility/ ). This section has its own submission website at https://mc.manuscriptcentral.com/e-videos .

## References

[1] Liu K Kaffes AJ Review article: the diagnosis and investigation of obscure gastrointestinal bleeding Aliment Pharmacol Ther 2011 34 416 423 10.1111/j.1365-2036.2011.04744.x 21692820

[2] Gerson LB Fidler JL Cave DR ACG clinical guideline: diagnosis and management of small bowel bleeding Am J Gastroenterol 2015 1265 1287 10.1038/ajg.2015.246 26303132

[3] Pasha SF Leighton JA Detection of suspected small bowel bleeding: challenges and controversies Expert Rev Gastroenterol Hepatol 2016 10 1235 1244 10.1080/17474124.2016.1207525 27366927

[4] Fidler JL Goenka AH Fleming CJ Small bowel imaging: computed tomography enterography, magnetic resonance enterography, angiography, and nuclear medicine Gastrointest Endosc Clin N Am 2017 27 133 152 10.1016/j.giec.2016.08.008 27908513

[5] Romãozinho JM Pontes JM Lérias C Dieulafoy’s lesion: management and long-term outcome Endoscopy 2004 36 416 420 10.1055/s-2004-814322 15100950

